# The polyphenol resveratrol promotes skeletal growth in mice through a sirtuin 1‐bone morphogenic protein 2 longevity axis

**DOI:** 10.1111/bph.14477

**Published:** 2018-09-18

**Authors:** Ming Zhao, Seon‐Yle Ko, I Ross Garrett, Gregory R Mundy, Gloria E Gutierrez, James R Edwards

**Affiliations:** ^1^ School of Medicine Tulane University New Orleans LA USA; ^2^ School of Dentistry Dankook University Cheonan Korea; ^3^ Department of Cellular and Structural Biology University of Texas Health Science Center at San Antonio and OsteoScreen Inc. San Antonio TX USA; ^4^ Botnar Research Centre, Nuffield Department of Orthopaedics, Rheumatology and Musculoskeletal Sciences University of Oxford Oxford UK

## Abstract

**Background and Purpose:**

The polyphenol resveratrol (RSV) exists in high quantities in certain foods (e.g. grapes and nuts). However, the capacity of RSV to confer physiological health benefits and a biological mechanism through which this might occur remains unclear.

**Experimental Approach:**

Aged, RSV‐treated (300 mg·kg^−1^·day^−1^) and genetically modified [endothelial NOS (eNOS^−/−^)] female mice were assessed using histomorphometric and μCT analysis. Alongside *in vivo* analysis, molecular siRNA knockdown and pharmacological manipulation of eNOS, BMP2 and sirtuin 1 (SIRT1) and functional cellular assays in an osteoblast cell line panel, explored the mechanism through which RSV might impact overall bone volume.

**Key Results:**

RSV promoted osteoblast activity and bone growth *in vivo*. RSV dose‐dependently and simultaneously increased alkaline phosphatase (ALP) and eNOS levels. Similarly, NO‐donor treatment increased ALP, runt homology transcription factor 2, BMP2 and stimulated bone formation, whilst eNOS‐deficient mice displayed a bone loss phenotype. Moreover, RSV‐induced increase in ALP and BMP2 expression was blocked in eNOS^−/−^ osteoblasts and by BMP‐inhibitor noggin. The longevity‐linked SIRT1 enzyme was positively regulated by RSV and SIRT1 deletion reduced eNOS, BMP2 and ALP. Like eNOS deletion, loss of SIRT1 blocked RSV‐induced osteoblast activity; however, SIRT1 levels remained unchanged in eNOS^−/−^ mice, indicating RSV activation of SIRT1 stimulates BMP2 release *via* eNOS. This signalling axis is supported by decreased SIRT1, eNOS and BMP2 confirmed in old versus young bone.

**Conclusions and Implications:**

These findings suggest a new mechanism of action in bone remodelling and the ageing skeleton, where RSV positively impacts bone homeostasis *via* SIRT1 activation of BMP2.

**Abbreviations**
ALP: alkaline phosphatase
BMD: bone mineral density
BMP2: bone morphogenic protein 2
eNOS: endothelial NOS
HDAC: histone deacetylase
MEM: minimal essential media
NAD+: nicotinamide adenine dinucleotide
RSV: resveratrol
Runx2: runt homology transcription factor 2
SIRT1: sirtuin 1

## Introduction

Diet impacts growth and ageing in all species, yet investigations exploring various dietary constituents as experimental medicines are limited in mechanism and translational impact, despite the potential for significant beneficial effects matching those offered by current pharmaceuticals.

http://www.guidetopharmacology.org/GRAC/LigandDisplayForward?ligandId=8741 (RSV; 3,4′,5‐trihydroxy‐transstilbene) is a plant polyphenol found in foods such as nuts, grapes and chocolate, and like other polyphenolic compounds (e.g. catechins in green tea extract) demonstrates various beneficial effects in preclinical and clinical studies for disorders such as cardiovascular disease (Smoliga *et al*., 2011; Sin *et al*., 2015a), diabetes (Szkudelski and Szkudelska, 2015) and inflammation (Huang *et al*., 2014; Conte *et al*., 2015).

Within the skeleton, dietary components are particularly important in regulating cellular metabolism and providing crucial constituents within bone (Sahni *et al*., 2015) particularly calcium. Bone is formed through the activities of osteoblasts. These mesenchymal‐derived, matrix‐producing cells lay down unmineralized osteoid in a highly regulated process, whilst simultaneously directing local mineralization through the release of matrix‐modifying enzymes, primarily alkaline phosphatase (ALP; de Gorter and ten Dijke, 2013).

RSV promotes osteoblast differentiation from mesenchymal stem cells *in vitro* (Zhou *et al*., 2009; Kupisiewicz *et al*., 2010; Shakibaei *et al*., 2012), reduces the formation of bone‐resorbing osteoclasts (Shakibaei *et al*., 2011) and may be protective in an experimental model of accelerated bone disease (Wang *et al*., 2017) suggesting that RSV supplementation may be beneficial for bone health to prevent the age‐related decline in functional integrity or as an experimental medicine in disorders of excessive bone destruction. However, the intracellular mechanisms activated by RSV stimulation remain complex and convoluted.

RSV is a powerful activator of the longevity‐linked sirtuin 1 (http://www.guidetopharmacology.org/GRAC/ObjectDisplayForward?objectId=2707) molecule (Howitz *et al*., 2003; Borra *et al*., 2005), the predominant member of the mammalian sirtuin longevity family of histone deacetylases (HDAC) (Michan and Sinclair, 2007). SIRT1 is a class III nicotinamide adenine dinucleotide (NAD+)‐dependent HDAC which also deacetylates non‐histone cytoplasmic substrate proteins, such as p53 and NF‐κB, to fine tune normal cell biology (Cheng *et al*., 2003; Salminen *et al*., 2008; Edwards *et al*., 2013). Through these activities, SIRT1 regulates important longevity‐related processes including apoptosis, cell survival, DNA repair and energy expenditure.

The NO‐generating enzyme http://www.guidetopharmacology.org/GRAC/ObjectDisplayForward?objectId=1249 (eNOS) is also identified as a SIRT1 substrate, and a downstream mediator of RSV action in endothelial cells (Ota *et al*., 2007). Moreover, the lifespan extension properties of caloric restriction might be regulated by a SIRT1‐eNOS axis (Nisoli *et al*., 2005). Within bone, eNOS is the most widely expressed isoform of the NOS family (Helfrich *et al*., 1997; MacPherson *et al*., 1999) where it is purported to mediate the anabolic effects of both oestrogen and mechanical strain on bone formation (Turner *et al*., 1996; Samuels *et al*., 2001), whilst our recent studies and others have shown that SIRT1‐deficiency hinders skeletal development, reduces peak bone mass and inhibits osteoblastic differentiation (Cohen‐Kfir *et al*., 2011; Edwards *et al*., 2013).

This translational study explores the *in vivo* mechanism through which RSV impacts bone formation, revealing an important stimulatory pathway in osteoblast formation and activity.

## Methods

### *In vivo* studies

All applicable international, national and institutional guidelines for the care and use of animals were followed. Animal studies are reported in compliance with the ARRIVE guidelines (Kilkenny *et al*., 2010) and were approved by the Institute of Animal Care and Use Committee office and conducted in accordance with the National Institutes of Health (NIH) Guide for the Care and Use of Laboratory Animals. RSV (ethanol stock in saline vehicle) was administered at 300 mg·kg^−1^·day^−1^ in the feeding chow, based upon our previous work and others (Tome‐Carneiro *et al*., 2013) aimed at achieving physiologically relevant circulating RSV levels, over a 10 week period to normal C57Bl/6 mice (female, 12 weeks old, 25–30 g avg. wt., *n* = 10) purchased from JAX lab (Maine, USA). The global eNOS knockout mice were generated by targeted deletion of part of the exon 12 sequence of the eNOS gene, as previously described (Aguirre *et al*., 2001). eNOS knockout mice were maintained as homozygotes on a C57BL6 background (female, *n* = 10). Normal female C57BL6 mice (as above) were aged within our facility and analysed at specific time points (3 months and 12 months, *n* = 10).

All mice were housed following standard LAR mouse housing protocols, with 4–5 mice per stainless steel cage in a room with a 12 h light/12 h dark cycle, kept at approx. 24°C with free access to food and water and killed by exsanguination and cervical dislocation. Randomization was used to assign animals to different experimental groups and to collect and process data, with analysis performed by investigators blinded to the treatment groups.

### X‐ray μ‐computed tomography (μCT)

The left tibia and femur were dissected and fixed in 10% phosphate‐buffered formalin for 48 h at 4°C. The microarchitecture of the proximal tibial and distal femoral metaphyses, within an area starting approximately 0.6 mm distal to the growth plate and extending 1.5 mm, was scanned at an isotropic voxel size of 16 μm using a μCT40 (ScanCo Medical, Basel, Switzerland). Reconstructed three‐dimensional (3D) images were obtained by semi‐manual contouring ~1000 slices in this area. Trabecular bone volume, trabecular number, trabecular thickness and trabecular separation or bone mineral density (BMD) were analysed using ScanCo μCT v.1.2 software.

### Histology

Tibia and femur samples were fixed in 10% phosphate‐buffered formalin and decalcified with 14% EDTA for 3 weeks, followed by paraffin‐embedding; 4 μm‐thick bone sections were stained with haematoxylin–eosin (H&E) to determine cellular distribution using Osteomeasure software (Osteometrics Atlanta, USA).

### Osteoblast culture

Primary bone marrow stromal cells were isolated from eNOS knockout mice and wild‐type control mice (female, 4 months, *n* = 5). The mice were killed by exsanguination and cervical dislocation, and the tibia and femur were removed and bone marrow cells were flushed out using an 18G syringe followed by filtration through 70 μm cell strainer with α‐minimal essential media (MEM) supplemented with 10% FBS. The bone marrow cells collected were cultured overnight to remove non‐adherent cells followed by a 5 day culture in osteogenic media prior to analysis and knockdown confirmed by qPCR (Figure S2A).

Mouse 2T3 (provided by Dr Stephen Harris, UTHSCSA, USA) and MC3T3‐E1 (ATCC, Teddington, UK) osteoblast cell lines were cultured with α‐MEM/FBS media. In mineralization studies, all cells were cultured in osteoblastogenic α‐MEM/FBS media containing 50 μg·mL^−1^ ascorbic acid, 8 mM β‐glycerol phosphate for 2–21 days, followed by von Kossa staining.

### siRNA knockdown

Osteoblasts (2T3 cells) were transiently transfected with mouse SIRT1 siRNA (Life Technologies/Ambion, Carlsbad, CA, USA, #s96766) or control, using Amine Transfection Agent (Life Technologies/Ambion, #AM4502), as optimized within our group; 24–48 h after siRNA transfection, significantly decreased SIRT1 expression levels were confirmed by quantitative PCR (Figure S2C).

### Alkaline phosphatase (ALP) activity

The 2T3, MC3T3‐E1 cells and primary osteoblasts were cultured in 48‐well plates in osteoblastogenic media supplemented with 2.5% FBS; 24–48 h after various treatments, cells were lysed with 0.05% Triton X‐100 buffer. Cell lysates were analysed for ALP activity in 96‐well plates. Briefly, 10 μL lysate was incubated with 90 μL fresh AMP solution containing p‐nitrophenyl phosphate substrate at 37°C for 30–60 min, following which 100 μL 0.5 N NaOH was added to stop the reaction. The plates were read spectrophotometrically at 405 nm. ALP activity was determined using a p‐nitrophenol standard curve and normalized to total cellular protein.

### SIRT1 activity

An assessment of overall SIRT1 activity was performed using the SIRT1 Fluorimetric Drug Discovery Kit (Enzo Life Sciences Exeter, UK) and following the manufacturer's instructions. In brief, samples were incubated with peptide substrate, and NAD+ in PBS 37°C, and the reaction was stopped with nicotinamide and the product visualized using a developing solution bound to the deacetylated lysine. Following 10 min incubation, the fluorophore was detected using a plate‐reading fluorimeter (excitation 360 nm and emission 450 nm). SIRT1 activity levels were normalized to total protein concentration.

### Calvarial organ culture

Parietal bone tissues were removed from the calvariae of 4‐ to 5‐day‐old male and female ICR Swiss mice bred within our facility, following cervical dislocation, and cultured on metal grids in BGJ (Biggers, Gwatkins, Judah) media with Fitton‐Jackson modification in the presence of NO donors or vehicle control for 4–7 days (*n* = 5). Following treatment, calvariae were fixed for 24 h in 10% phosphate‐buffered formalin and decalcified in 14% EDTA overnight. Calvariae were embedded in paraffin, and 6 μm sections cut and stained with haematoxylin and eosin. The culture media was collected to measure ALP activity as described above.

### Luciferase reporter assay

Primary osteoblasts null for eNOS were cultured in 24‐well plates and co‐transfected with a bone morphogenic protein (http://www.guidetopharmacology.org/GRAC/LigandDisplayForward?ligandId=4881) promoter reporter construct, −2712/+165‐Luc, and a pRL‐TK vector (Promega, Madison, WI, USA) using the Lipofectamine™ LTX and Plus Reagent Transfection Reagent (Invitrogen, Carlsbad, USA) according to the manufacturer's protocol. Cells were treated with resveratrol at 10 μM for 24–48 h. Luciferase activity in cell lysates was assessed using the Dual‐Luciferase® Reporter Assay System (Promega, # E1910) and a luminometer according to the manufacturers' protocols. The relative reporter firefly luciferase activity was normalized to renilla luciferase activity of the control pRL‐TK vector.

### Real time PCR

Total RNA was extracted from cells or grounded long bones under liquid nitrogen with TRI Reagent® RNA Isolation Reagent. Total RNA was purified and reverse transcribed into cDNA. Quantitative real time PCRs were performed through Applied Biosystems' TaqMan® Gene Expression Assays with the cDNA templates and primers Mm011658521‐A1, Mm00435206‐g1, Mm01962382_s1, Mm00501578_m1 and Mm03413826_mH, specifically for mouse SIRT1, eNOS, BMP2, runt homology transcription factor 2 (Runx2) and osteocalcin mRNAs, on 7900 Real Time PCR System (Applied Biosystems, Foster City, USA). Eukaryotic 18S rRNA was detected using VIC/MGB Probe (4319413E); GAPDH served as an endogenous control.

### Nitrite and eNOS protein assay

The levels of nitrite in the osteoblast supernatant following cell lysis and centrifugation were determined by a colorimetric assay using a modified Griess Reagent. Briefly, 1× Griess Reagent was mixed with equal volumes of sample. The absorbance at 540 nm was read after 15 min. The nitrite concentrations were calculated using a standard curve. Similarly, eNOS total protein and phosphorylated eNOS at a specific serine residue (phospho‐Ser^1176^) were quantified in treated cell lysates using an established ELISA Kit (Creative Diagnostics, Shirley, USA) following the manufacturer's instructions.

### BMP2 ELISA

After treatment with NO donors for 24–48 h, the concentration of BMP2 within the media was determined using a Quantikine BMP2 ELISA (R&D Systems, Minneapolis, USA). Recombinant human BMP2 (hBMP2; R&D Systems Inc.) was used as a standard.

### Data and statistical analysis

The data and statistical analysis comply with the recommendations on experimental design and analysis in pharmacology (Curtis *et al*., 2015). Data were analysed using GraphPad Prism 5 and presented as mean ± SEM. *In vitro* studies were performed on *n* = 5 independent cultures, unless otherwise stated. Sample size estimates for *in vivo* studies are based on a survey of data from published research and preliminary studies. To detect a twofold change between experimental groups, power analysis using probability of type I error α = 0.05; probability of type II error β = 0.20, along with previous laboratory data, indicated that *n* = 10 mice per group would be required in each experiment to detect a significant change with an α error of 5% and a power of 0.93. The Mann–Whitney *U*‐test was used for comparisons made between two groups of data that are not normally distributed. Comparison between multiple groups was made with the one‐way ANOVA followed by Dunnett's test. For all one‐way ANOVAs, *post hoc* tests were run only if F achieved *P* < 0.05 and there was no significant variance inhomogeneity. In all cases, *P* values < 0.05 were considered significant.

### Materials

Materials and reagents were purchased and used as indicated above, from Sigma‐Aldrich (Gillingham, UK), unless otherwise stated, and including molecular Ambion reagents (ThermoFisher Scientific, Hemel Hempstead, UK). The botanical nutrient resveratrol (3,4′,5‐trihydroxy‐transstilbene) was purchased from Sigma‐Aldrich and nitric oxide donors NOC22 (Spermine NONOate, #567703) and SNP (sodium nitroprusside dihydrate, #567538) were purchased from Millipore/Calbiochem (Watford, UK).

### Nomenclature of targets and ligands

Key protein targets and ligands in this article are hyperlinked to corresponding entries in http://www.guidetopharmacology.org, the common portal for data from the IUPHAR/BPS Guide to PHARMACOLOGY (Harding *et al*., 2018), and are permanently archived in the Concise Guide to PHARMACOLOGY 2017/18 (Alexander *et al*., 2017).

## Results

### Resveratrol stimulates osteoblast differentiation and increases bone mass *in vivo*

**Figure 1:**
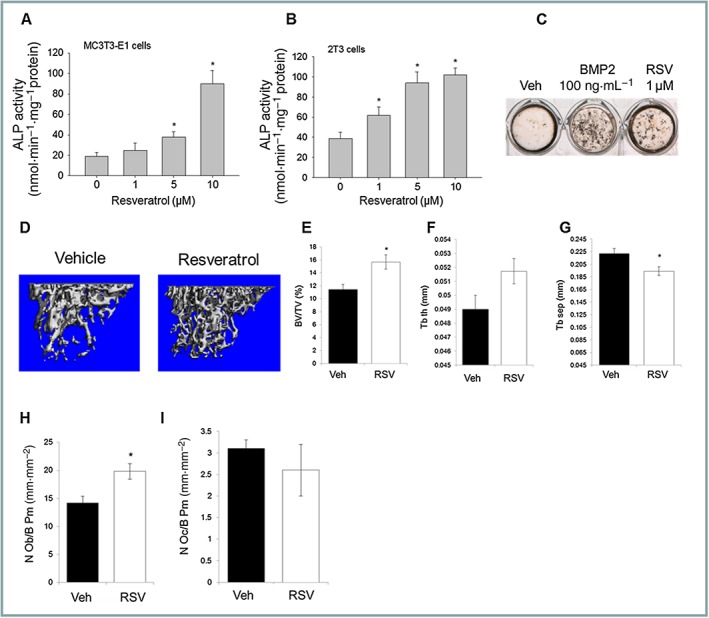
RSV stimulates osteoblasts *in vitro* and bone formation *in vivo*. ALP activity of MC3T3‐E1 (A) and 2T3 (B) osteoblast cells treated with RSV (1–10 μM) or vehicle for 2 days (*n* = 5); normalization to total cell protein. (C) Mineralized matrix formation rates of 2T3 osteoblasts cultured in osteogenic media and RSV (1 μM, *n* = 5) or vehicle over 2 weeks (von Kossa staining). Normal C57Bl/6 mice (2 month) treated with resveratrol (300 mg·kg^−1^·day^−1^) in prepared chow, versus control chow over 10 weeks (*n* = 10). Bone volume (BV/TV) and trabecular parameters assessed in dissected tibia by μCT scanning (D–G), and cellular distribution by histomorphmetric analysis following H&E or TRAP staining (H–I) (*P < 0.05 vs. vehicle).

**Figure 2:**
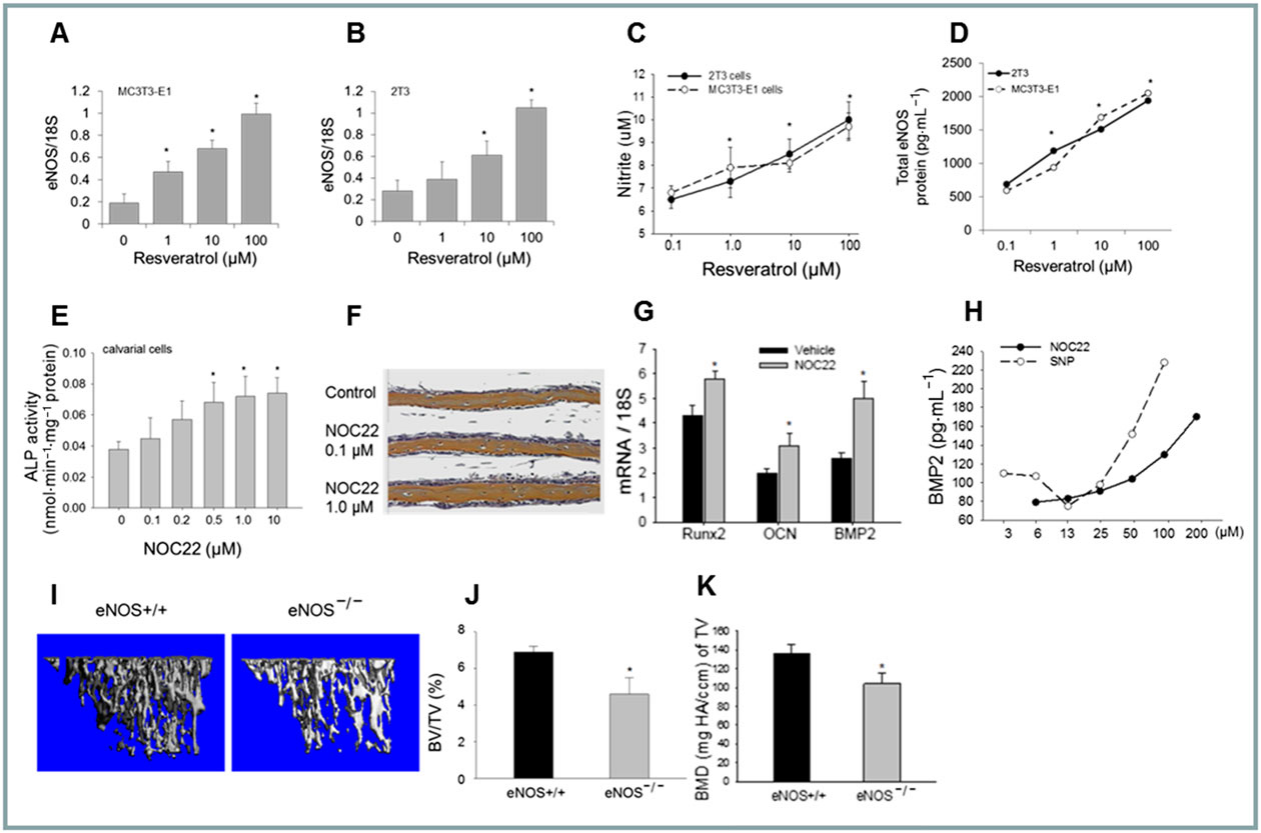
eNOS and NO‐donors stimulate bone growth. eNOS mRNA expression in MC3T3 (A) and 2T3 (B) cells treated with RSV (1–100 μM) or vehicle for 24 h (*n* = 5) determined by real time PCR. Nitrite (NO_2_^−^) levels (C) or total eNOS protein (D) from 2T3 or MC3T3 cells treated with RSV (1–100 μM) or vehicle (*n* = 5) for 48 h, determined by colorimetric assay. ALP levels from primary osteoblasts treated with NO‐donor (NOC22, 0.1–10 μM) or vehicle for 4 days (*n* = 5) normalized to total cell protein (E). Calvariae from newborn mice cultured with NO‐donor (NOC22, 0.1–1 μM) or vehicle control for 4 days (*n* = 5) processed for histology and H&E staining (F). mRNA expression of osteoblast marker genes Runx2, osteocalcin (OCN) and BMP2 determined in 2T3 cells treated with NOC22 (1.0 μM) or vehicle for 24 h (*n* = 5) by real time PCR (G). BMP2 protein levels assessed in conditioned media from NO‐donor‐treated osteoblasts (NOC22 or SNP, 3–200 μM, 48 h, *n* = 5), by ELISA (with rhBMP2 as standard) (H). The μCT analysis of dissected tibia from homozygous eNOS knockout (^−/−^) or wild‐type (^+/+^) control mice (4 month, *n* = 10) (I) and trabecular bone volume (J) and BMD (K) analysis (*P < 0.05 vs. vehicle; *P < 0.01 vs. wild‐type control animals).

Treatment of established MC3T3 (Figure 1A) or 2T3 osteoblast cell lines (Figure 1B) with RSV dose‐dependently increased alkaline phosphatase, a clinical marker of bone‐forming activity, and stimulated mineralization *in vitro* (Figure 1C). Moreover, normal C57Bl/6 female mice treated with RSV (300 mg·kg^−1^·day^−1^) over 10 weeks (*n* = 10) showed increased bone volume (Figure 1D,E) and trabecular parameters (Figure 1F,G) following analysis by μCT scanning. Histomorphometric analysis of bone cell distribution indicated that this was brought about by a significant increase in osteoblast numbers in the RSV‐treated group, compared to control (Figure 1H) with no significant change in osteoclast numbers (Figure 1I). These *in vivo* and *in vitro* results demonstrate that the botanical nutrient resveratrol is an effective anabolic agent for bone mass and it functions, at least in part, by promoting osteoblast differentiation.

### eNOS and NO‐donors stimulate bone growth

RSV has been suggested to induce physiological effects such as vasodilatation through an up‐regulation of eNOS (Wallerath *et al*., 2002; Breen *et al*., 2012). In MC3T3 (Figure 2A) and 2T3 (Figure 2B) osteoblast cell lines treated with RSV, eNOS gene expression was dose‐dependently upregulated, concomitantly with soluble levels of nitrite product (Figure 2C) and total eNOS protein levels (Figure 2D). However, no significant change in phosphorylation status was induced by RSV treatment when compared to the overall increase in total eNOS levels (expressed as phospho eNOS/total eNOS, Figure S1).

In a similar manner to RSV treatment, the NO‐donor compound NOC22 increased alkaline phosphatase levels in primary osteoblasts *in vitro* (Figure 2E) and in an *ex vivo* bone formation model (Figure 2F). Real time‐PCR analysis indicated NO‐donor treatment of osteoblasts up‐regulated osteogenic genes Runx2, OCN and BMP2 (Figure 2G) and increased soluble BMP2 release (Figure 2H). Importantly, μCT analysis of long bones from genetically modified mice deficient in the eNOS gene (Figure S2A) demonstrated a significant decrease in overall bone volume (36%) (Figure 2I,J) and bone mineral density (22%) (Figure 2K).

### eNOS‐SIRT1 axis is necessary for pro‐osteogenic effects of RSV

**Figure 3:**
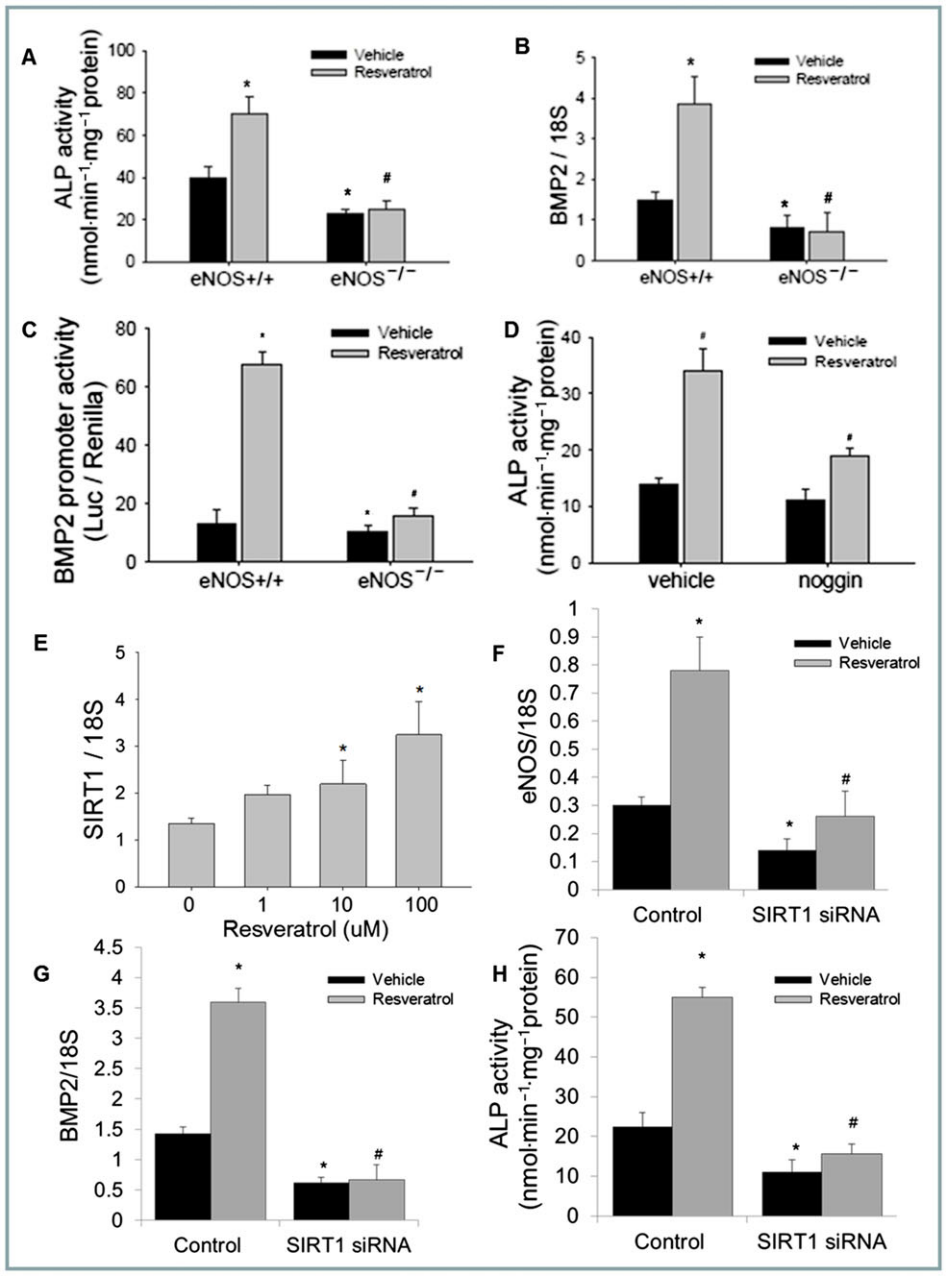
The eNOS‐SIRT1 axis is necessary for the pro‐osteogenic effects of RSV. Different effects on ALP levels (A) BMP2 gene expression (qPCR, B) and promoter activity (C) in primary osteoblasts from eNOS knockout (^−/−^) or wild‐type (^+/+^) control mice (*n* = 5) treated with RSV (5 μM) or vehicle (24 h). RSV‐induced ALP levels (5 μM) in the presence of the BMP inhibitor noggin (500 ng·mL^−1^, 48 h, *n* = 5), normalized to total protein (D). SIRT1 gene expression in 2T3 osteoblasts following RSV (1–100 μM) treatment, quantified by real time PCR (E), along with eNOS (F) and BMP2 (G) mRNA levels transfected with SIRT1 siRNA or scramble control (*n* = 5) with and without RSV treatment (5 μM). RSV‐induced ALP levels (5 μM) in the presence of SIRT1 (or scrambled control) siRNA (H) (*n* = 5) (*P < 0.05 vs. vehicle treated WT/scrambled ctrl; ^#^ *P* < 0.001 vs. RSV‐treated WT/scrambled ctrl).

Given the positive effects of RSV treatment on eNOS and NO expression, combined with our observations indicating loss of eNOS reduced bone whilst NO generation stimulates osteoblast activity, we investigated whether eNOS deficiency impairs RSV effects in bone‐forming osteoblasts. As expected, RSV treatment of primary osteoblasts isolated from normal mice increased alkaline phosphatase levels. However, RSV failed to increase alkaline phosphatase levels in osteoblasts from eNOS‐deficient mice (Figure 3A). Similarly, the RSV‐induced increase in BMP2 promoter activity and mRNA expression were ablated in the absence of eNOS (Figure 3B,C). Interestingly, the RSV‐induced increase in alkaline phosphatase was reduced by the endogenous BMP inhibitor noggin (Figure 3D).

The ageing‐related SIRT1 factor has been shown to regulate bone cell biology and may underlie age‐related bone loss (Cohen‐Kfir *et al*., 2011; Edwards *et al*., 2013). As many effects of RSV are thought to be mediated through SIRT1, we examined whether SIRT1 was altered in osteoblasts following RSV treatment. An increase in SIRT1 expression was confirmed by qPCR at 10 and 100 μM of RSV (Figure 3E) along with increased SIRT1 activity (Figure S2B). However, knockdown of SIRT1 using an established validated siRNA that reduced SIRT1 expression and activity (Figure S2B,C) led to a reduction in eNOS and reduced the capacity of RSV (5 μM) to stimulate eNOS (Figure 3F) or SIRT1 expression (Figure S2C). Furthermore, SIRT1 knockdown also reduced BMP2 gene expression and inhibited both the RSV‐induced (5 μM) increase in BMP2 expression (Figure 3G) and alkaline phosphatase (Figure 3H).

### Ageing decreases bone volume and eNOS‐BMP2 expression

**Figure 4:**
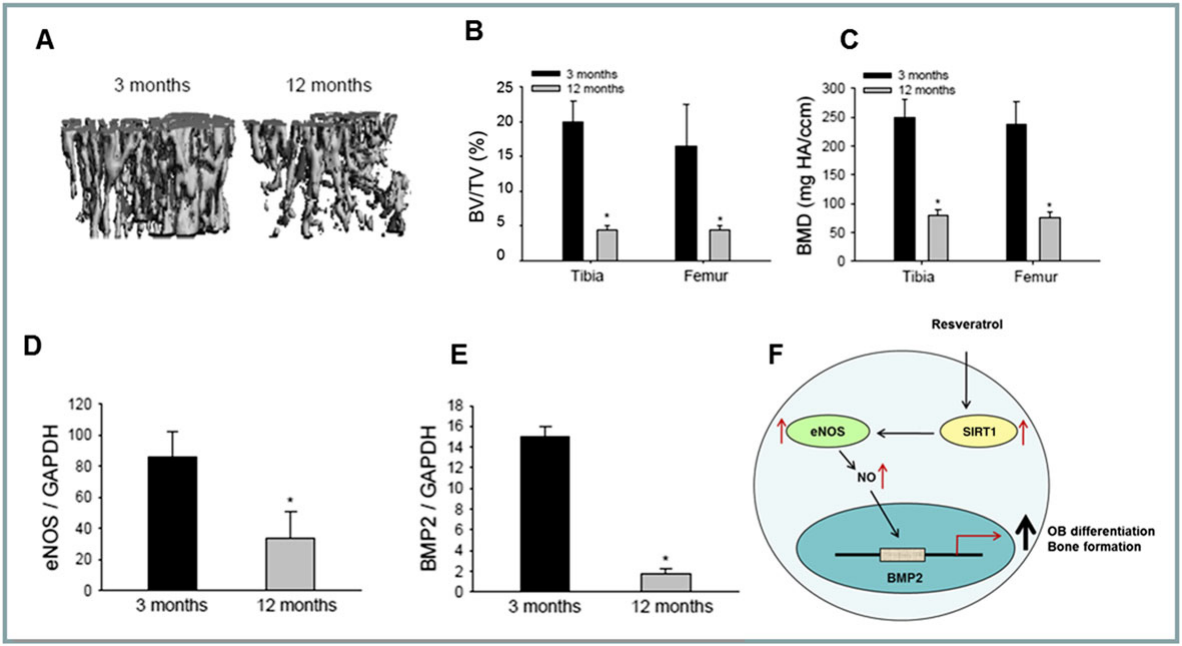
Ageing decreases bone volume and eNOS‐BMP2 expression. μCT analysis of tibia of young (3 month) or aged (12 month) mice (*n* = 10) (A) determining BV/TV (B) and BMD (C). Gene expression changes (real time PCR) of eNOS (D), BMP2 (E) in ageing long bones (*n* = 10). Proposed mechanism of RSV action within osteoblasts (F) (*P < 0.05).

Age‐related bone loss was confirmed in the long bones of 12‐month‐old female mice, compared to younger 3 month animals, by μCT analysis, showing decreased bone volume (Figure 4A,B) and BMD (Figure 4C). Previously, our studies and others have shown that SIRT1 expression declines in ageing tissue, including bone (Edwards *et al*., 2013). In support of an upstream role for SIRT1 in ageing bone, 12‐month‐old mice also displayed a significant decrease in eNOS (Figure 4D) and BMP2 expression (Figure 4E).

## Discussion

These studies suggest that RSV exerts an anabolic effect within the skeleton to stimulate osteoblast activity and promote bone formation, brought about following activation of SIRT1, triggering an up‐regulation of eNOS and BMP2 (Figure 4F).

RSV is reported to activate SIRT1 in many species and cell systems, including *Caenorhabditis elegans*, drosophila, yeasts and human cells, and contributes to the beneficial life‐extending properties of RSV (Howitz *et al*., 2003; Borra *et al*., 2005). A variety of potential pathways may be activated following RSV treatment and account for such effects, including intracellular factors such as Ca^2+^ (Zhang *et al*., 2013), ERK1/2 (Bellaver *et al*., 2015) and FoxO proteins (Sin *et al*., 2015b). In bone cells, elevated Ca^2+^ signalling is known to control osteoblast growth and differentiation (Zayzafoon, 2006) but also lead to increased and sustained stimulation of the master osteoclastogenic regulator NFATc1, to enhance bone resorption (Negishi‐Koga and Takayanagi, 2009), a feature not observed in our *in vivo* model suggesting RSV‐stimulated Ca^2+^ signalling does not occur in this system.

Similarly, FoxO1 and FoxO3a can be activated by RSV‐SIRT1 and also regulate eNOS activity, where deletion of FoxO1 and FoxO3a abolished the effect of RSV on eNOS expression in endothelial cells (Xia *et al*., 2013) and deletion of FoxO1,‐3 and ‐4 blocked the anti‐resorptive effect of novel SIRT1 activating compounds structurally distinct from RSV (Bartell *et al*., 2014; Kim *et al*., 2015). As reported, osteoclast‐specific effects were not observed in our RSV‐treated model where positive changes in bone‐forming osteoblasts appeared to mediate the physiological response to RSV treatment, suggesting different pharmacological activation of SIRT1 might trigger alternate downstream effectors. However, osteoblastogenesis might also be altered by the SIRT1‐FoxO axis (Iyer *et al*., 2014) and where FoxO activation of BMP2 in anti‐cancer treatments (Su *et al*., 2007) might suggest an overlapping role in RSV‐SIRT1 induced BMP2 activation in osteoblasts also. It is clear that additional factors may have the potential to intersect and modulate the signalling axis proposed, including the post‐translational modification of such proteins, to modify the effectiveness of RSV stimulation of BMP2 *via* SIRT1‐eNOS activation.

SIRT1 is an upstream activator of eNOS in other cell systems, particularly endothelial cells (Mattagajasingh *et al*., 2007; Ota *et al*., 2007), and eNOS is highly expressed in osteoblasts (Helfrich *et al*., 1997; MacPherson *et al*., 1999). We found that not only RSV but SIRT1 directly stimulated the activity of eNOS, increasing NO in osteoblasts. The physiological significance of this pathway is highlighted by the increased bone volume stimulated by RSV treatment *in vivo*, and the loss of bone volume observed in SIRT1‐deficient animals (Edwards *et al*., 2013) or following eNOS deletion. This study also provides evidence that NO production is up‐regulated by RSV. Consistent with our findings that NO‐releasing compounds stimulate osteoblast differentiation and bone formation, NO‐donors also antagonize ovariectomy‐ or corticosteroid‐induced bone loss in rodents (Wimalawansa *et al*., 1997; Hukkanen *et al*., 2003) and improve fracture healing (Corbett *et al*., 1999; Diwan *et al*., 2000). Similarly, recent work suggests RSV might also be protective in an experimental model of osteoporosis in rats (Wang *et al*., 2017), supporting our hypothesis that RSV might increase overall bone mass under normal conditions. However, whether such findings are truly translatable to human physiology remain to be determined where for example bioavailability of RSV and lifestyle factors may enhance or diminish the effects observed in rodent models (Tome‐Carneiro *et al*., 2013; Berman *et al*., 2017). It remains a misconception that sufficient levels of RSV are achieved through a normal standard diet alone. Further controlled studies in human subjects investigating alternate RSV preparations, strong supplementation or managed dosing options for administration, along with the extent and rate at which RSV is taken up by the body in a physiologically active form, remain necessary.

Resveratrol is an established antioxidant and offers protection against the damaging cellular effects of ROS which are known to accumulate in ageing tissues (Truong *et al*., 2018) and shift the commitment of mesenchymal stem cells towards an adipogenic phenotype at the expense of osteoblasts by decreasing SIRT1 function (Lin *et al*., 2018), though conversely, ROS might also activate BMP2 and osteoblast differentiation (Mandal *et al*., 2011). We therefore cannot exclude the possibility that altered ROS levels following RSV‐treatment might lead to changes in BMP2 and osteoblast formation. Interestingly, elevated BMP2 is itself associated with increased oxidative stress and pro‐inflammatory signals in other tissue types, which may abrogate long‐term effects of our final observation *in vivo* (Csiszar *et al*., 2006; Chen *et al*., 2015).

Resveratrol has also been reported to regulate osteoclast activity *in vitro*. However, the reported effects of resveratrol on osteoclast formation are not consistent, varying with different cell sources (Boissy *et al*., 2005; Voronov *et al*., 2005). Work from our group and others using SIRT1‐conditional and ‐germ line knockout mice supports the notion that SIRT1 is a genetic determinant of bone mass and acts in a cell‐autonomous manner in both osteoblasts and osteoclasts (Cohen‐Kfir *et al*., 2011; Edwards *et al*., 2013). This suggests that RSV may exert different effects, through alternate pathways, in various cell types, and that its primary mechanism of action within bone is *via* increased osteoblast activation and enhanced bone formation, as indicated by *in vivo* observations. However, whether this approach might prove effective in an ageing bone environment remains to be determined. Osteoblasts in old bone are suggested to be less numerous and less active than those in a mature skeleton, whilst other cell populations are increased in number (e.g. adipocytes). The observed anabolic effects of RSV in our study might be altered by the changes in cellular composition and tissue structure of aged bone. The use of an established experimental approach for the testing of bone targeted therapies in this study, where skeletally mature animals are utilized as test models, might be complemented by further *in vivo* testing in old mice if disease‐free, aged mice of similar size and bone density could be reliably sourced.

Despite the common worldwide use of various anti‐resorptive agents to prevent bone loss, the availability of anabolic agents capable of stimulating new bone growth within the aged skeleton remains limited. The concept that low dose dietary components might induce an accumulative beneficial effect over time, to prevent the decrease in formation and activity of osteoblasts seen in old bone, suggests a significant additional approach through the prophylactic management of bone loss.

These data provide evidence for the use of RSV, to protect against bone loss by stimulating the pro‐osteogenic factor BMP2. Loss of SIRT1 levels with increasing age are implicated in the progression of age‐related disorders in many tissues. Maintaining SIRT1 levels through the use of such naturally occurring factors as RSV may slow the onset of ageing and increase health span.

## Author contributions

M.Z., G.R.M., G.E.G. and J.R.E. planned the study. M.Z., S.Y.K., I.R.G. and J.R.E. performed *in vitro* and *in vivo* experimentation. M.Z. and J.R.E. prepared the manuscript.

## Conflict of interest

The authors declare no conflicts of interest.

## Declaration of transparency and scientific rigour

This http://onlinelibrary.wiley.com/doi/10.1111/bph.13405/abstract acknowledges that this paper adheres to the principles for transparent reporting and scientific rigour of preclinical research recommended by funding agencies, publishers and other organisations engaged with supporting research.

## Supporting information


**Figure S1** RSV treatment and eNOS protein levels. Protein lysates from MC3T3 and 2 T3 osteoblast cells treated with RSV showed increased phospho‐eNOS (A) and no significant change as compared to RSV effects on total eNOS levels (B) by ELISA.
**Figure S2** Gene knockdown and activity status. Knockdown of eNOS in cells isolated from genetically modified and control mice was confirmed by qPCR (A). Protein lysates from RSV‐treated (5 μM) 2 T3 cells showed increased activity of SIRT1 enzyme while SIRT1 siRNA transfection decreased SIRT1 activity (B). Furthermore, RSV‐induced stimulation of SIRT1 activity was reduced in SIRT1 siRNA transfected cells only, with a non‐significant change compared to vehicle treatment (B). SIRT1 expression following siRNA or control transfection confirmed by qPCR, with and without^+/−^ RSV treatment (5 μM)(C)(*P < 0.05 *vs* vehicle treated ctrl; ^##^ *P* < 0.01 *vs* WT (^+/+^) cells; ^#^ *P* < 0.001 *vs* RSV‐treated scrambled ctrl, ^###^ *P* < 0.001 *vs* scrambled ctrl).
