# Supplementary figures and images for: The polyphenol resveratrol promotes skeletal growth in mice through a sirtuin 1‐bone morphogenic protein 2 longevity axis
Source: Br J Pharmacol. 2018 Sep 18;175(21):4183–92. doi: 10.1111/bph.14477 (PMC6177622; doi:10.1111/bph.14477)

Suppl. Figure 1

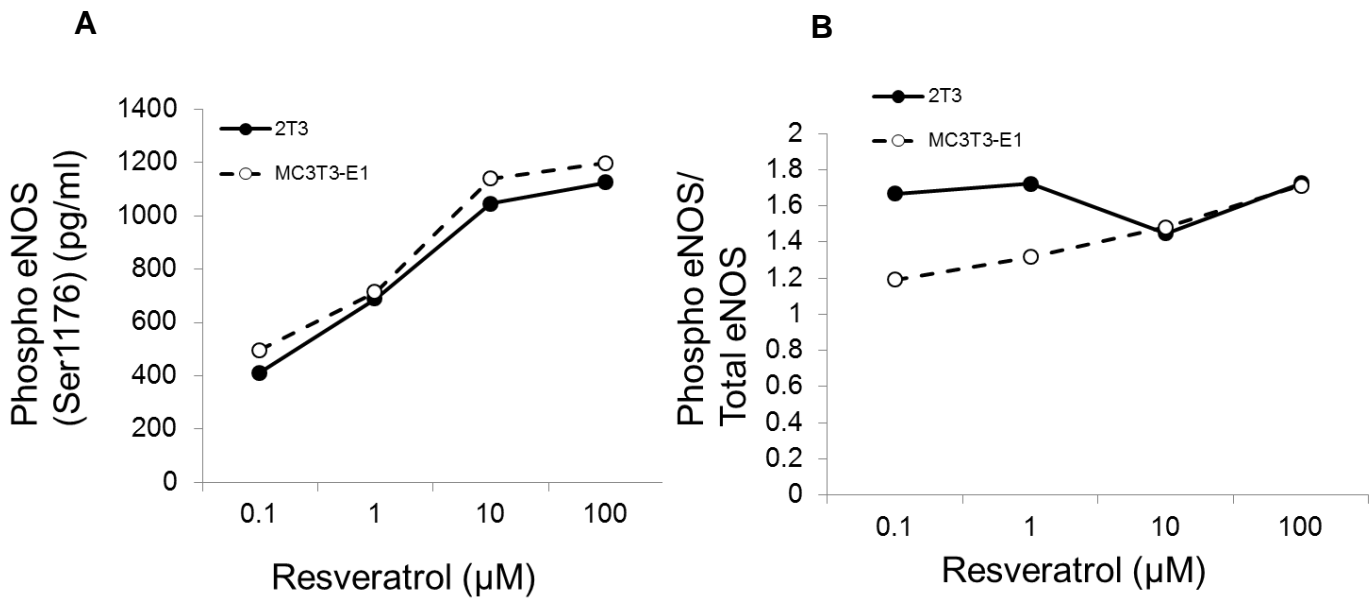

Suppl. Figure 2

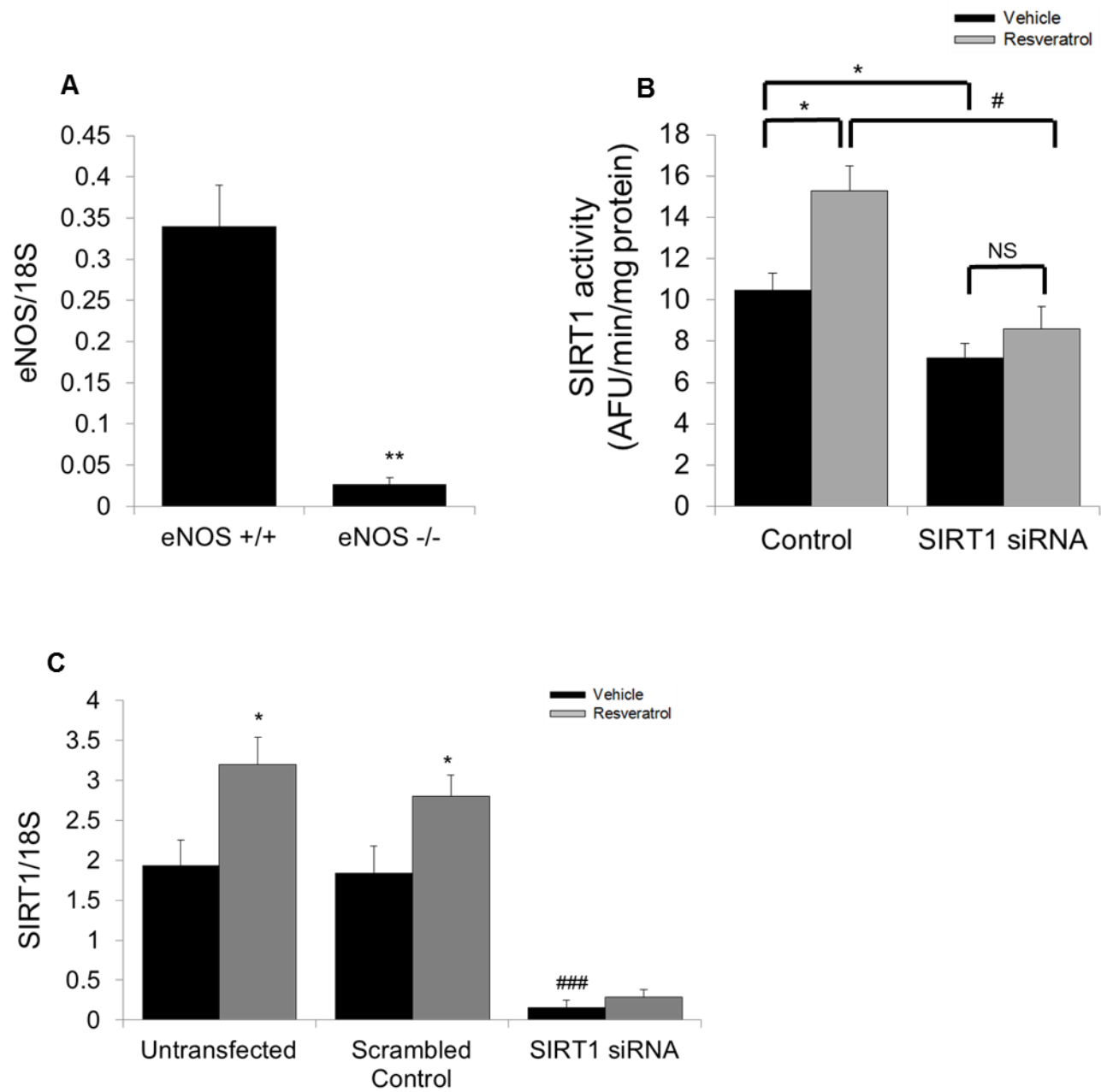

Supplement: Supplementary file 1 — Figure S1 RSV treatment and eNOS protein levels. Protein lysates from MC3T3 and 2 T3 osteoblast cells treated with RSV showed increased phospho‐eNOS (A) and no significant change as compared to RSV effects on total eNOS levels (B) by ELISA. Figure S2 Gene knockdown and activity status. Knockdown of eNOS in cells isolated from genetically modified and control mice was confirmed by qPCR (A). Protein lysates from RSV‐treated (5 μM) 2 T3 cells showed increased activity of SIRT1 enzyme while SIRT1 siRNA transfection decreased SIRT1 activity (B). Furthermore, RSV‐induced stimulation of SIRT1 activity was reduced in SIRT1 siRNA transfected cells only, with a non‐significant change compared to vehicle treatment (B). SIRT1 expression following siRNA or control transfection confirmed by qPCR, with and without+/− RSV treatment (5 μM)(C)(*P < 0.05 vs vehicle treated ctrl; ## P < 0.01 vs WT (+/+) cells; # P < 0.001 vs RSV‐treated scrambled ctrl, ### P < 0.001 vs scrambled ctrl). [file BPH-175-4183-s001.pdf]
